# Stacking Control by Molecular Symmetry of Sterically Protected Phthalocyanines

**DOI:** 10.3390/molecules25235552

**Published:** 2020-11-26

**Authors:** Ryota Kudo, Masahiro Sonobe, Yoshiaki Chino, Yu Kitazawa, Mutsumi Kimura

**Affiliations:** 1 Department of Chemistry and Materials, Faculty of Textile Science and Technology, Shinshu University, Ueda 386-8567, Japan; 18fs410j@shinshu-u.ac.jp (R.K.); 19fs414e@shinshu-u.ac.jp (M.S.); 17st109g@shinshu-u.ac.jp (Y.C.); 2 Research Initiative for Supra-Materials (RISM), Interdisciplinary Cluster for Cutting Edge Research (ICCER), Shinshu University, Ueda 386-8567, Japan; yu_kitazawa0311@shinshu-u.ac.jp

**Keywords:** phthalocyanine, molecular symmetry, stacking, crystal, melting points

## Abstract

The synthesis and characterization of two phthalocyanine (Pc) structural isomers, **1** and **2**, in which four 2,6-di(hexyloxy)phenyl units were attached directly to the 1,8,15,22- or 1,4,15,18-positions of the Pc rings, are described. Both Pcs **1** and **2** exhibited low melting points, i.e., 120 and 130 °C respectively, due to the reduction in intermolecular π-π interaction among the Pc rings caused by the steric hindrance of 2,6-dihexyloxybenzene units. The thermal behaviors were investigated with temperature-controlled polarizing optical microscopy, differential scanning calorimetry, powder X-ray diffraction, and absorption spectral analyses. Pc **1**, having C_4h_ molecular symmetry, organized into a lamellar structure containing lateral assemblies of Pc rings. In contrast, the other Pc **2** revealed the formation of metastable crystalline phases, including disordered stacks of Pcs due to rapid cooling from a melted liquid.

## 1. Introduction

**Figure 1:**
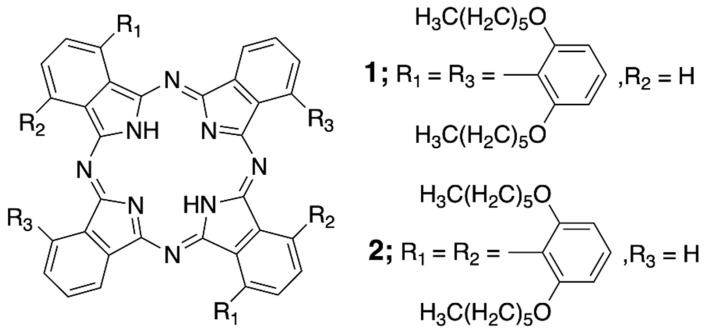
Chemical structures of **1** and **2**.

Molecular-based functional modules have been constructed on the basis of a bottom-up approach for the designed molecular units [1,2,3]. These molecule units can be organized into hierarchical architectures through the control of the molecular shape, intermolecular noncovalent interactions, polarity of solvents, temperatures, and interactions with solid substrates [1,2,3]. The ordered orientation of the π-conjugated molecular units within the supramolecular architectures enabled highly efficient charge transportation, along with the molecular assemblies and the tuning of light absorption [4]. Phthalocyanines (Pcs) have also been used as the molecular component for the construction of supramolecular architectures [5]. Planar and disk-shaped π-conjugated Pc rings can be stacked into columnar assemblies through their strong π-π interactions. In particular, Pcs attached with flexible alkyl chains have been investigated as major thermotropic discotic liquid crystalline materials; their phase segregation between the Pc core and surrounding alkyl chains allow the creation of well-organized structures of one-dimensional columnar stacks while also keeping long-range ordering of Pc stacks within the column [6]. So far, systematic structural exploration of liquid-crystalline Pcs has been achieved by changing central metals and alkyl chains to tune the stacking modes of Pcs. However, the high viscosity and melting temperature of Pc-based liquid crystals make them poorly suited to forming large-area and uniform organized states without defects. In this study, we designed two structural isomers, **1** and **2**, having different molecular symmetries (**1**: C_4h_, **2**: D_2h_) for the substitution of four 2,6-dihexyloxybenzene units at the nonperipheral positions of the Pc ring to control the stacking modes of Pcs in condensed solids (Figure 1) [7]. The attachment of two hexyloxy groups at positions 2 and 6 of the phenyl units in **1** and **2** can prevent the rotation of phenyl units around the Pc rings, and the 2,6-dihexyloxybenzene units lie outside of the plain of the Pc rings. The steric hindrance of 2,6-dihexyloxybenzene around Pc rings may change the stacking modes through the reduction of the intermolecular π-π interactions among Pc rings. Both **1** and **2** exhibited low melting points as compared with the reported alkyl-modified Pcs, and formed large-area uniform domains due to cooling from isotropic liquids, whereas **1**, possessing C_4h_ symmetry, showed a simple reversible thermotropic transition between crystal and liquid phases, and **2**, having D_2h_ symmetry, displayed a slow crystallization process depending on the cooling speed.

## 2. Results and Discussion

**Scheme 1:**
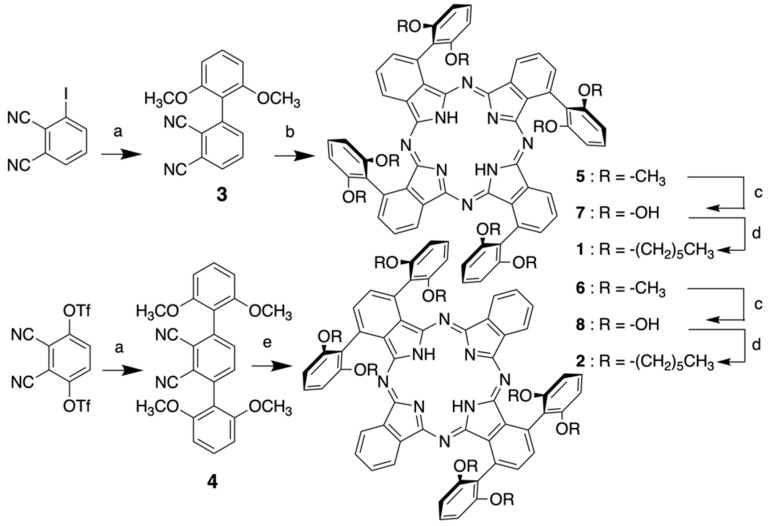
Synthetic procedures of **1** and **2**. Regents and conditions: (**a**) 2,6-dimethoxyphenyl boronic acid, Pd(OAc)_2_, SPhos, CsCO_3_, 1,4-dioxane, 100 °C, 20 h. (**b**) LiO(CH_2_)_5_CH_3_, n-hexanol, 157 °C, 5 h. (**c**) BBr_3_, CH_2_Cl_2_, 0 °C to rt, 8 h. (**d**) n-hexylbromide, K_2_CO_3_, DMF, 60 °C, 3 days. (**e**) phthalonitrile, LiO(CH_2_)_5_CH_3_, n-hexanol, 160 °C, 4 h.

Two Pcs, **1** and **2**, having 2,6-dihexyloxybenzene substituents at four α-positions of the Pc ring, were synthesized from 3-(2′,6′-di(methoxy)phenyl)phthalonitrile **3** by mixing phthalonitrile and 3,6-di(2′,6′-bis(methoxy)phenyl))phthalonitrile **4** (Scheme 1) [8,9]. Two precursors, **3** and **4**, were prepared from 3-iodophthalonitrile or 3,6-ditriflate phthalonitrile and 2,6-dimethoxyphenylboronic acid by the Suzuki-Miyaura coupling reaction using 2-dicyclohexylphosphino-2′,6′-methoxybiphenyl (SPhos) as a ligand in 90 and 40% yields, respectively. After cyclic tetramerization of **3** or two phthalonitriles (**4** and phthalonitrile) by LiOC_6_H_13_ in n-hexanol, eight methoxy groups were cleaved by BBr_3_ to give 1,8,15,22- and 1,4,15,18-tetra(2′,6′-dihydroxyphenyl)phthalocyanines **7** and **8**. Finally, two target compounds, **1** and **2**, were obtained through the Williamson ether reaction between **7** or **8** and 1-bromohexane in the presence of K_2_CO_3_. All compounds were fully characterized by ^1^H-NMR, ^13^C-NMR, atmospheric pressure chemical ionization-time-of-flight (APCI-TOF) mass, and matrix-assisted laser desorption ionization-time-of-flight (MALDI-TOF) mass spectroscopies.

**Figure 2:**
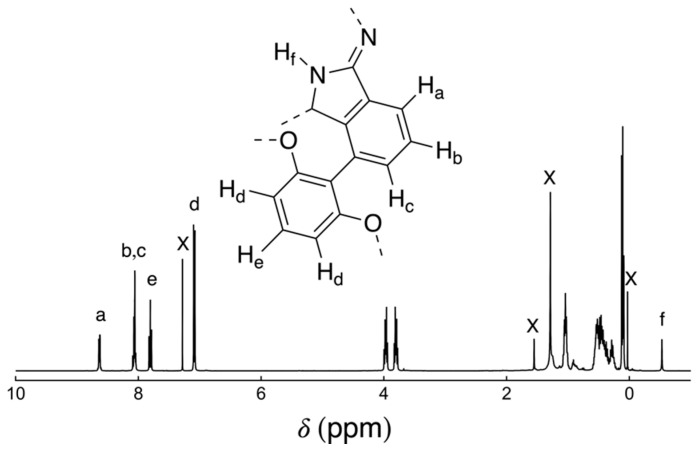
^1^H-NMR spectrum of **1** in CDCl_3_. The signals for residue solvents and the internal standard TMS are denoted as X.

Cyclic tetramerization of 3-substituted phthalonitriles usually produces a mixture of four regioisomers having different molecular symmetries (D_2h_, C_2v_, C_4h_, and C_s_). The reaction mixture during the tetramerization of **3** at 157 °C showed a simple ^1^H-NMR spectrum, suggesting the formation of a single symmetrical isomer. Several attempts for the selective formation of *C*_4h_ isomers from 3-substituted phthalonitriles have been demonstrated [10,11,12]. Although the size of the methoxy groups in **3** was relatively small, as compared with the reported substituents for the successful selective formation of *C*_4h_ isomers, the restriction of the dihedral angle between the two benzene rings in **3** prevented the formation of the other D_2h_, C_2v_, and C_s_ isomers under a high reaction temperature by keeping a good yield (48%). The methoxy groups in **5** were exchanged with hexyloxy groups by cleavage with BBr_3_ and subsequent ether formation. We also tried to prepare **1** from 3-(2′,6′-di(hexyloxy)phenyl)phthalonitrile; however, its yield was quite low (less than 1%) under the same reaction conditions as those used for **3**. The large steric hindrance of hexyloxy groups would diminish the approach of using four phthalonitriles to form the Pc ring. The resulting Pc **1** can dissolve in many solvents except for alcohol and water, and it showed sharp, simple, and assignable ^1^H-NMR spectra under a 1 × 10^−2^ mol/dm^3^ concentration at 25 °C (Figure 2). The wide solubilities in the solvents and the sharp ^1^H-NMR peaks at a high concentration indicate good dispersion as monomeric species without the formation of intermolecular aggregates in solvents, due to the steric hindrance of 2,6-dihexyloxybenzene substituents at the four α-positions of the Pc ring in **1**.

**Figure 3:**
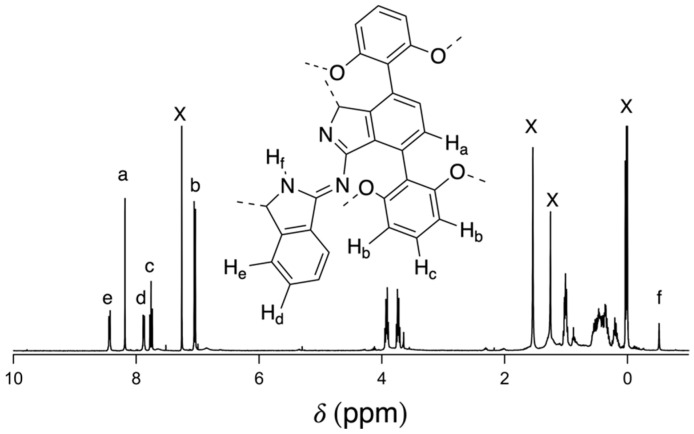
^1^H-NMR spectrum of **2** in CDCl_3_. The signals for residue solvents and the internal standard TMS are denoted as X.

**Figure 4:**
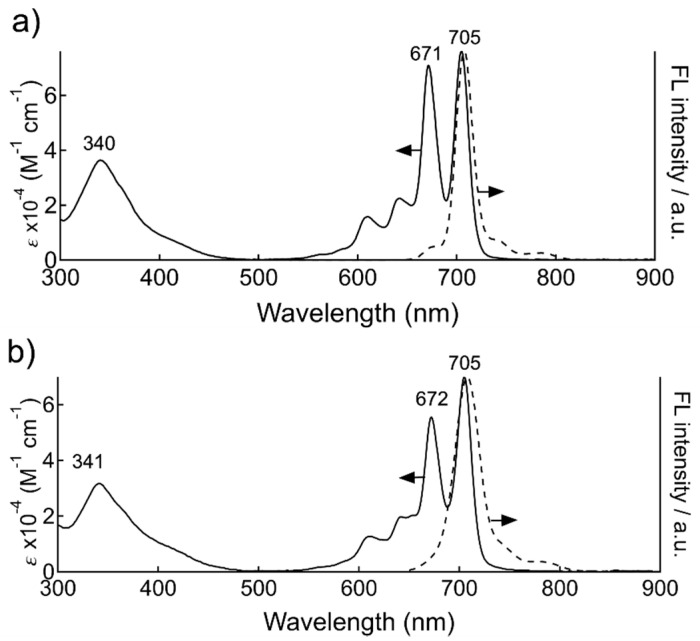
Absorption (solid line) and fluorescence (dashed line) spectra of **1** (**a**) and **2** (**b**) in CH_2_Cl_2_.

**Figure 5:**
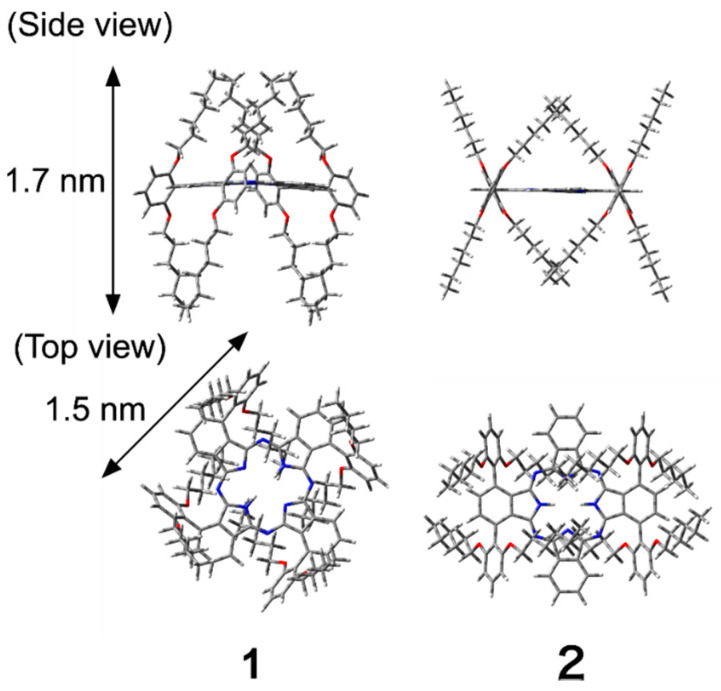
Molecular dimensions of **1** and **2** optimized by B3LYP/6-31G* level of Gaussian 16 (Gaussian, Inc., Wallingford, CT, USA).

Various asymmetric Pc rings (AAAB, ABAB, and AABB types) have been prepared through several approaches such as the statical mixing, the ring-expansion of subphthalocyanines, and the control of the steric repulsion of two different phthalonitriles [13]. The selective preparation of the ABAB-type Pcs has been shown to construct multicomponent molecular architectures, having linear arrangements. Torres et al. succeeded in the efficient synthesis of ABAB-type Pcs by using 3,6-di(3′,5′-bis(trifluoromethyl)phenyl)phthalonitrile [14]. Since the attachment of two phenyl groups in the 3 and 6 positions of phthalonitriles is not enough to avoid self-condensation [15], the introduction of additional bulky substituents into the side benzenes is key to inhibiting the formation of distorted Pcs and AABB-type Pcs. We expect that the introduction of two methoxy groups at the 2 and 6 positions of the side benzenes will enhance the selectivity for the formation of the ABAB-type Pc. First, a Pc ring was not obtained by the tetramerization of **2** under the reported condensation conditions [15], revealing that the attachment of two 2,6-dihexyloxybenzene substituents at positions 3 and 6 of phthalonitrile interfered with the self-condensation of **2** into the distorted Pc ring. The ABAB-type Pc **2** was synthesized as a structural isomer of **1** by mixing phthalonitrile and **4**. The MALDI-TOF mass spectrum of the reaction mixture during the mixed condensation showed three mass peaks at *m*/*z* 526, 798, and 1070 g/mol, corresponding to Li_2_Pc, AAAB containing one isoindole (**B**) functionalized with two bulky bis(2,6-dimethoxy)phenyl units, and ABAB (or AABB) types. Compound **6** with *m*/*z* 1058 g/mol was successfully separated from the mixture by column chromatography and preparative HPLC. The ^1^H-NMR spectrum of **2** exhibited only five peaks in the aromatic region, indicating the formation of an ABAB-type Pc (Figure 3) [7]. Furthermore, the proton signal for the two inside protons in the Pc ring was located at −0.51 ppm, suggesting no distortion of the Pc ring. Figure 4a,b show the absorption and fluorescence spectra of **1** and **2** in CH_2_Cl_2_. A comparison of the spectral shapes and peak positions for both spectra of **1** and **2** showed excellent agreement, revealing that the difference in molecular symmetries for **1** and **2** did not affect the electronic conditions of their Pc rings. Thus, structural isomers **1** and **2** with C_4h_ and D_2h_ molecular symmetries were successfully synthesized. Eight flexible hexyloxy chains in **1** and **2** were placed on both sides of the Pc plane and partially covered with a Pc skeleton (Figure 5).

**Figure 6:**
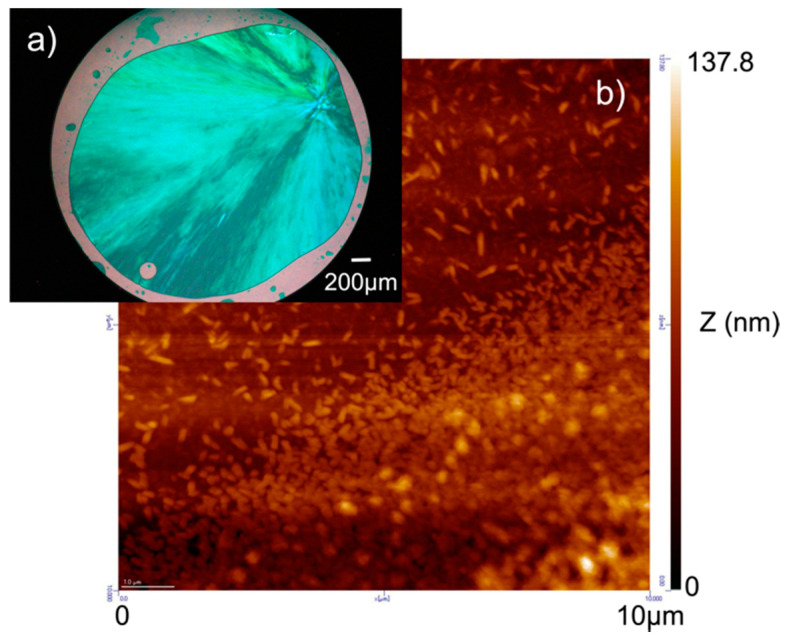
(**a**) Polarized optical microscope and (**b**) AFM images (substrate: mica) of **1** film prepared from cooling from isotropic liquid.

Thermogravimetric analyses of **1** and **2** showed that the weight-losses started at around 300 °C under N_2_ atmosphere, indicating their thermal stability up to 300 °C (Figures S1 and S2). The thermal phase transition behaviors of **1** and **2** were checked by using a temperature-controlled polarizing optical microscope (TPOM) and differential scanning calorimetry (DSC) analysis. Compounds **1** and **2** melted at 120 and 130 °C, respectively, and the liquids showed no birefringence under POM observation. Liquid-crystalline Pcs with flexible alkyl chains at peripheral positions of the Pc core possessed two transitions, that is: a crystalline phase to a mesophase, and then a mesophase to an isotropic liquid [6]. At the first transition between the crystalline phase to the mesophase, the alkyl side chains in the liquid crystalline Pcs started to melt while maintaining the stack of the Pc molecules. The π-π stacking interaction in the Pc stacks could break at the second transition from the mesophase to the isotropic liquid. In contrast, the DSC profile of **1** displayed only one reversible transition at 121 and 70 °C upon heating and cooling runs at 10 °C/min (Figure S3). This transition enthalpy (58.0 kJ/mol) roughly agreed with the reported melting enthalpy of side hexyl chains in 2,3,9,10,16,17,24-octa(hexyloxy)phthalocyanine [16]. According to the results of TPOM and DSC for **1**, the observed transition can be assigned to the melting point from crystal to isotropic liquid. Compound **1**, possessing relatively short alkyl chains, exhibited a significantly lowed melting point compared with the reported values for Pcs with hexyl chains [17]. On cooling from the isotropic liquid, a birefringence texture appeared for the whole domain of **1** through crystallization (Figure 6a). An atomic force microscope (AFM) image of a thin film of **1** showed assemblies of short needles 30 nm in width (Figure 6b).

**Figure 7:**
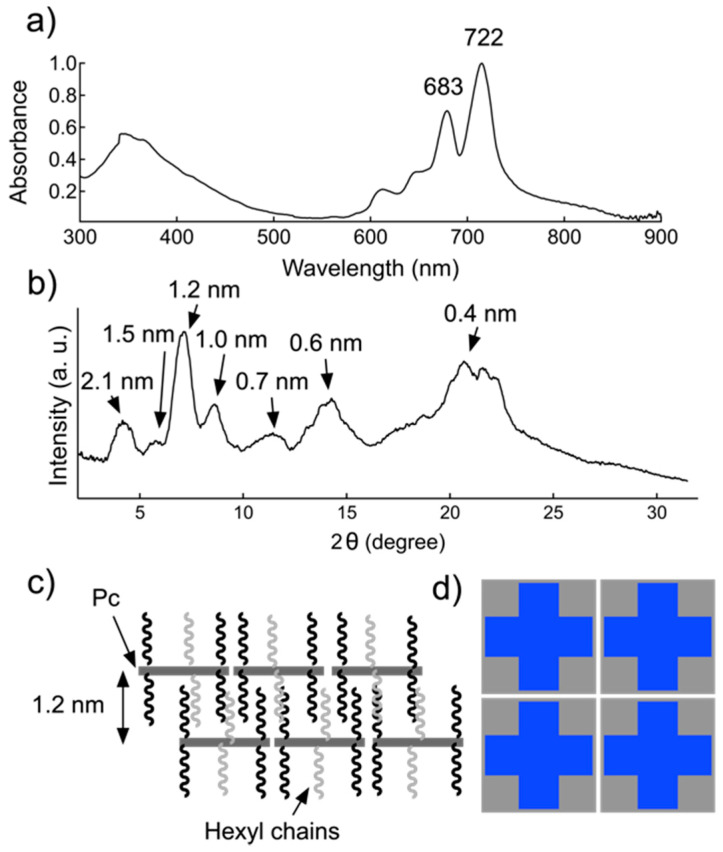
(**a**) Absorption spectrum and (**b**) XRD pattern of **1** film at room temperature. Schematic representation of the proposed structure of the crystalline phase of **1**: (**c**) view along the molecular z-axis, (**d**) view along the normal on the Pc xy-plane.

The molecular arrangement of Pcs in the crystalline phase were analyzed on the basis of the temperature-controlled absorption spectra and X-ray diffraction patterns (XRD). The absorption spectral shape for the Q bands of Pcs is known to be sensitive to the local environment of individual molecules [16]. Figure 7a shows the absorption spectrum of the **1** film prepared by cooling from an isotropic liquid to room temperature. The Q band for the **1** film at room temperature was slightly broadened, and the Q band position was red-shifted from 705 nm in solution to 722 nm. The emission peak position was also shifted to a longer wavelength by 19 nm. The spectrum above the melting point was sharpened, and the maximum of the Q band peaks was shifted to a shorter wavelength. According to the molecular exciton theory [18], the red shift of the Q band suggests the parallel alignment of Pcs having inline transition dipoles. Whereas many planar Pcs show blue shift in the Q band to support the formation of columnar aggregates, through π-π interaction among Pcs, the bulky 2,6-dihexyloxybenzene substituents in **1** served to prevent the formation of stacks of Pc molecules. The structures of the crystalline phase and isotopic liquid of **1** were determined by XRD measurements at room temperature and at 140 °C (Figure 7b). The XRD pattern of **1** at 140 °C displayed only a broad and diffuse halo around 0.48 nm, which could be ascribed to the liquid-like disorder in alkyl chains. Upon cooling from 140 °C to room temperature, several broad reflections appeared at 2θ = 3–25 degree. The three reflections emerging at 1.2 nm, 0.6 nm, and 0.4 nm in a space ratio of 1:1/2:1/3 indicated that the crystalline **1** contained a lamellar structure. Judging from the simulated molecular dimensions of **1** by Gaussian 16 (Figure 4), the observed distance of 1.2 nm was shorter than the estimated molecular length of **1** with fully elongated hexyloxy chains, i.e., 1.7 nm. This implied that the lamellar layers may have originated from the head to tail interdigitated structure of the flexible hexyl chains on both sides of the Pc plain (Figure 7c). The other reflections at 1.5 and 2.1 nm coincided with the simulated side and diagonal lengths of the square α-substituted Pc plane, suggesting the formation of lateral assemblies of Pc rings in the lamellar structure (Figure 7d). However, the weak and broad XRD reflections for **1** suggest a short molecular ordering in the crystalline phase.

**Figure 8:**
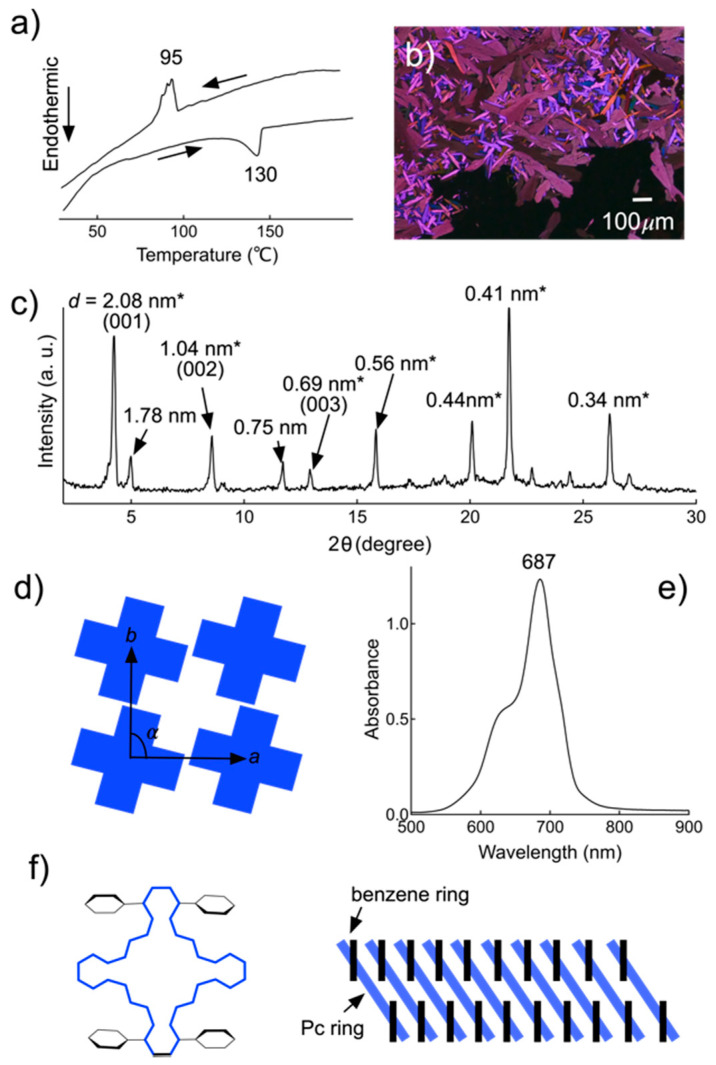
(**a**) DSC thermogram of **2** film during the first cooling and the second heating scans at 2 °C/min. (**b**) POM image of **2** at 93 °C cooling from 130 °C at 2 °C/min. (**c**) XRD pattern of **2** film at room temperature prepared by slow cooling from isotropic liquid at 2 °C/min. * shows diffraction peaks for one monoclinic lattice (a = 2.31 nm, b = 2.06 nm, c = 0.41 nm, α = 94°, β = γ = 90°). The other set of peaks can be indexed to a monoclinic lattice having different lattice constant (a = 1.81 nm, b = 1.77 nm, c = 0.83 nm, α = 93°, β = γ = 90°). (**d**) Schematic representation of the proposed structure of the crystalline phase of **2** from the view along the molecular xy-axis. (**e**) Absorption spectrum of **2** film at room temperature prepared by slow cooling from isotropic liquid at 2 °C/min. (**f**) Schematic representation of the proposed structure of the crystalline phase of **2** from the view along the molecular z-axis.

Figure 8a shows the DSC profiles of ABAB-type **2** upon heating and cooling runs at 2 °C/min. During the first heating runs from 30 to 200 °C, **2** exhibited one endothermic peak at 130 °C. Subsequent cooling at 2 °C/min showed an exothermic peak at 95 °C, and the transition enthalpies for the heating and cooling processes were comparable. When **2** was cooling from an isotropic liquid at 2 °C/min, birefringent short needles appeared at 95 °C under TPOM (Figure 8b), and sharp and intense peaks in the XRD measurement were detected (Figure 8c). According to an analysis of the XRD pattern, **2** crystal contained two crystal structures possessing different monoclinic lattices of Pcs. The XRD peaks, as indicated by * in Figure 8c, can be indexed according to a monoclinic lattice with Pcs with a unit cell with the parameters a = 2.31 nm, b = 2.06 nm, c = 0.41 nm, and α = 94° (Figure 8d). The center–center distance of **2** along the a-axis agreed with the simulated molecular length with elongated hexyl chains. The difference between the a- and b-axes suggested the tilting of the Pc cores within the stacks. The Pc cores were probably tilted with respect to the c-axis, and the tilted angle of the cores in the stacks could be calculated to be 34° [19]. Two sharp XRD peaks at d = 0.34 and 0.41 nm suggested a long-range ordered stacking of Pcs and benzene rings within the crystals. Furthermore, the appearance of several sharp reflections in the wide-angle region suggested a dense packing of the hydrocarbon chains in the crystals. The absorption spectrum of crystalline **2** was broadened, and the maximum of the Q band was blue-shifted to 687 nm, indicating the formation of a cofacial stack of Pc molecules (Figure 8e). Thus, the Pc rings in **2** could form long-range ordered stacks while avoiding the steric hindrance of 2,6-dihexyloxybenzene substituents attached to the 1, 4, 15 and 18 positions of the Pc rings due to slow cooling from an isotropic liquid (Figure 8f).

**Figure 9:**
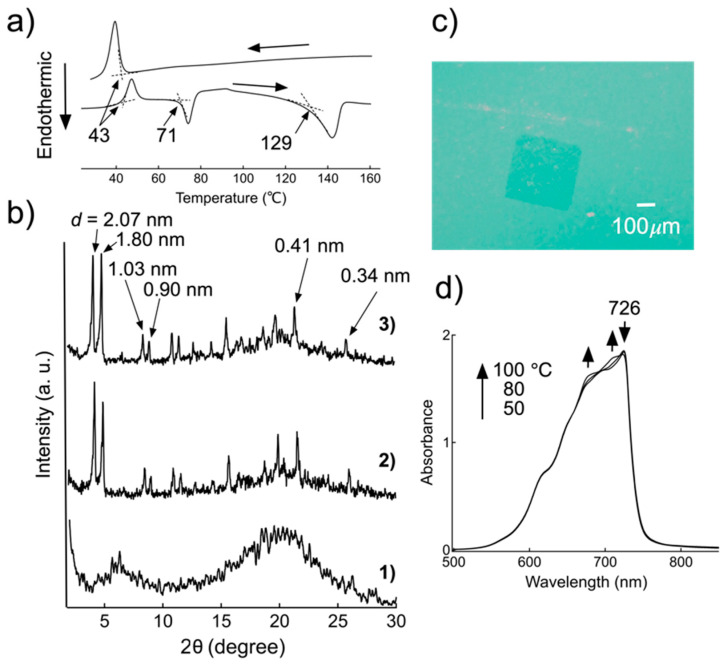
(**a**) DSC thermogram of **2** film during the first cooling and the second heating scans at 20 °C/min. (**b**) XRD pattern of **2** film at the first cooling run at 45 °C (**1**), the second heating run at 60 (**2**) and 100 °C (**3**) at 20 °C/min. (**c**) optical image of **2** at 60 °C. (**d**) Absorption spectrum of **2** film during the second heating run at 50, 80, and 100 °C.

In the DSC cooling run of **2** from an isotropic liquid at 20 °C/min, an exothermic peak was observed at 43 °C, which was lower than that obtained at 2 °C/min (Figure 9a). The XRD patterns for **2** exhibited only a broad halo (Figure 9b), and no birefringence was observed through TPOM. These results revealed that **2** was in a supercooled state due to rapid cooling from an isotropic liquid [20]. The second heating run from 10 °C revealed one exothermic crystallization peak at 43 °C and two endothermic peaks at 71 and 129 °C. The XRD patterns at 60 and 100 °C were comparable, and the intensities for the two peaks at 0.34 and 0.41 nm were weaker than those for the crystalline sample prepared by slower cooling at 2 °C/min (Figure 9b). Dark square domains including a latticed pattern were grown above 43 °C, supporting a square arrangement of Pcs in crystal (Figure 9c) [21]. The **2** film exhibited a red-shifted absorption band, observed at 726 nm (Figure 9d). Upon heating above the first endothermic peak, new shoulder peaks at 687 and 704 nm appeared, indicating a change in the Pc stacking mode. From these results, we assumed that the rapid temperature change could form metastable crystalline phases by diminishing the long-range ordered stacking of Pcs.

## 3. Conclusions

In summary, the crystalline structures of two structural isomers, **1** and **2**, possessing different molecular symmetries, were investigated. The spatial overlapping of hexyloxy groups onto the π-surface of the Pc rings resulted in a significant decrease in the melting points, and large homogeneous domains were formed by cooling from the melting points. C_4h_ isomer **1** organized into a layered structure by two-dimensional assembly without π-π stacking among Pcs. In contrast, D_2h_ isomer **2** was able stack in the crystalline phase, and the ordering in the stacks depended on the cooling speeds from the isotropic liquid. Since Pc crystals have been used in several organic devices such as organic thin film transistors and organic photovoltaic cells due to their highly efficient electron or charge transportations [22,23], the thin films consisting of Pc crystals prepared by solution processes and thermal annealing have good potential as molecular components for such devices. Research on applying these Pcs to organic devices is in progress.

## Supplementary Materials

Figure S1: TGA profile of 1 under N_2_ (scan rate 10 °C/min), Figure S2: TGA profile of 2 under N_2_ (scan rate 10 °C/min), Figure S3: DSC thermogram of 1 during the first cooling (top line) and the second heating scans (bottom line) at 10 °C/min.

## References

[1] Kato T. (2002). Self-Assembly of Phase-Segregated Liquid Crystal Structures. Science.

[2] Wu J., Pisula W., Müllen K. (2007). Graphenes as Potential Material for Electronics. Chem. Rev..

[3] Aida T., Meijer E.B., Stupp S.I. (2012). Functional Supramolecular Polymers. Science.

[4] Hoeben F.J.M., Jonkheijm P.P., Meijer E.B., Schenning A.P.H.J. (2005). About Supramolecular Assemblies of π-Conjugated Systems. Chem. Rev..

[5] Lezonoff C.C., Lever A.B.P. (1989). Phthalocyanines: Properties and Applications.

[6] Wöhrle T., Wurzbach I., Kirres J., Kostidou A., Kapernaum N., Litterscheidt J., Haenle J.C., Staffeld P., Baro A., Giesselmann F. (2016). Discotic Liquid Crystals. Chem. Rev..

[7] Fukuda T., Homma S., Kobayashi N. (2005). Deformed Phthalocyanines: Synthesis and Characterization of Zinc Phthalocyanines Bearing Phenyl Substituents at the 1-, 4-, 8-, 11-, 15-, 18-, 22-, and/or 25-Positions. Chem. A Eur. J..

[8] Chino Y., Nakanishi T., Kimura M. (2020). A near-infrared fluorescent phthalocyanine liquid developed through controlling intermolecular interactions. New J. Chem..

[9] Al-Raqa S.Y. (2006). Synthesis, photochemical and photophysical properties of novel unsymmetrically substituted zinc(II) phthalocyanines. J. Porphyr. Phthalocyanines.

[10] Brewis M., Clarkson G.J., Humberstone P., Makhseed S., McKeown N.B. (1998). The synthesis of some phthalocyanines and naphalocyanines derived from sterically hindered phenols. Chem. Eur. J..

[11] Bian Y., Li L., Dau J., Cheng D.Y.Y., Li R., Ma C., Ng D.K.P., Kobayashi N., Jiang J. (2004). Synthesis, structure, spectroscopic properties, and electrochemistry of (1,8,15,22-tetrasubstitiuted phthalocyaninato)lead complexes. Inorg. Chem..

[12] Ranta J., Kumpulainen T., Lemmetyinen H., Efimov A. (2010). Synthesis and characterization of monoisomeric 1,8,15,22-substituted (A3B and A2B2) phthalocyanines and phthalocyanine-fullerene dyes. J. Org. Chem..

[13] Mack J., Kobayashi N. (2011). Low Symmetry Phthalocyanines and Their Analogues. Chem. Rev..

[14] Fazio E., Jaramillo-Garcia J., De La Torre G., Torres T. (2014). Efficient Synthesis of ABAB Functionalized Phthalocyanines. Org. Lett..

[15] Kobayashi N., Fukuda T., Ueno K., Ogino H. (2001). Extremely Non-Planar Phthalocyanines with Saddle or Helical Conformation: Synthesis and Structural Characterizations. J. Am. Chem. Soc..

[16] van Nostrum C.F., Picken S.J., Schouten A.-J., Nolte R.J.M. (1995). Synthesis and supramolecular chemistry of novel liquid crystalline crown ether-substituted phthalocyanines: Toward molecular wires and molecular ionoelectronics. J. Am. Chem. Soc..

[17] Cherodian A.S., Davies A.N., Richardson R.M., Cook M.J., McKeown N.B., Thomson A.J., Feijoo J., Ungar G., Harrison J. (1991). Mesogenic Behaviour of some 1,4,8,11,15,18,22,25-Octa-alkylphthalocyanines. Mol. Cryst. Liq. Cryst..

[18] Kasha M., Rawls H.R., El-Bayoumi M.A. (1965). The exciton model in molecular spectroscopy. Pure Appl. Chem..

[19] Ohmori M., Nakano C., Higashi T., Miyano T., Tohnai N., Fujii A., Ozaki M. (2016). Single crystal growth and X-ray structure analysis of non-peripheral octahexyl phthalocyanine. J. Cryst. Growth.

[20] Lu F., Jang K., Osica I., Hagiwara K., Yoshizawa M., Ishii M., Chino Y., Ohta K., Ludwichowska K., Kurzydłowski K.J. (2018). Supercooling of functional alkyl-π molecular liquids. Chem. Sci..

[21] Ramananarivo M.F., Higashi T., Ohmori M., Fujii A., Ozaki M. (2015). Single crystal growth in spin-coated films of polymorphic phthalocyanine derivative under solvent vapor. APL Mater..

[22] Dong S., Bao C., Tian H., Yan D., Geng Y., Wang F. (2012). ABAB-Symmetric Tetraalkyl Titanyl Phthalocyanines for Solution Processed Organic Field-Effect Transistors with Mobility Approaching 1 cm^2^ V^−1^ s^−1^. Adv. Mater..

[23] Miyake Y., Shiraiwa Y., Okada K., Monobe H., Hori T., Yamasaki N., Yoshida H., Cook M.J., Fujii A., Ozaki M. (2011). High carrier mobility up to 1.4 cm^2^∙V^−1^∙s^−1^ in non-peripheral octahexyl phthalocyanine. Appl. Phys. Exp..

