# Supplementary material for: Stacking Control by Molecular Symmetry of Sterically Protected Phthalocyanines
Source: Molecules. 2020 Nov 26;25(23):5552. doi: 10.3390/molecules25235552 (PMC7730234; doi:10.3390/molecules25235552)
Supplement: Supplementary file 1 [file molecules-25-05552-s001.pdf]

Supporting Information

Stacking Control by Molecular Symmetry of Sterically Protected Phthalocyanines

Ryota Kudo[1], Masahiro Sonobe[1], Yoshiaki Chino[1], Yu Kitazawa[2], and Mutsumi Kimura[1,2*]

[1]Department of Chemistry and Materials, Faculty of Textile Science and Technology, Shinshu University, Ueda 386-8567, Japan, [2]Research Initiative for Supra-Materials (RISM), Interdisciplinary Cluster for Cutting Edge Research (ICCER), Shinshu University

General. NMR spectra were recorded on a Bruker AVANCE 400 FT NMR spectrometer at 399.65 MHz and 100.62 MHz for ^{1}H and ^{13}C in $CDCl_3$ solution. Chemical shifts are reported relative to internal tetramethylsilane. Absorption and fluorescence spectra were measured on a SHIMAZU UV-2600 and a JASCO spectrophotometer FP-8600, respectively. MALDI-TOF mass spectra were obtained on a Bruker Microflex spectrometer with dithranol as matrix. Mass spectra with electrospray ionization were obtained on a Bruker Daltonics micrOTOFII. Polarizing optical microscopic observation was carried out with polarizing optical microscope (NIKON ECLIPSE LV100ND) equipped with a hot stage (METTLER TOLEDO FP82HT Hot Stage). Thermogravimetric analyses (TGA) were carried out with Seiko Instruments EXSTAR TG/DTA6200 under nitrogen gas flow at scan rate 10 ^{o}C/min. Differential scanning calorimetry (DSC) was carried out with Hitachi High-tech Science DSC7020 with liquid nitrogen cooling under nitrogen gas flow. X-ray diffractogram was recorded with Rigaku X-ray diffractometer RINT-Ultima / S2K (Cu K_{α} source).

All chemicals were purchased from commercial supplies used without further purification. Column chromatography was performed with activated alumina (Wako, 200mesh) or silica gel (Wakogel C-200). Recycling preparative gel permeation chromatography was carried out by a JAI recycling preparative HPLC using $CHCl_3$ as an eluent. Analytical thin layer chromatography was performed with commercial Merck plates coated with silica gel 60 F_{254} or aluminum oxide 60 F_{254}.

Synthesis (Scheme 1)

3-(2’,6’-dimethoxyphenyl)phthalonitrile **3**: A flask containing 3-iodophthalonitrile (300

mg, 1.18 mmol), 2,6-dimethoxybenzeneboronic acid (280 mg, 1.53 mmol), $CsCO_3$ (1.53 g, 4.72 mmol), $Pd(OAc)_2$ (5.3 mg, 0.024 mmol) and SPhos (39 mg, 0.095 mmol) was filled with Ar. 9.0 mL of dry dioxane was added to the mixture with syringe through septum. The mixture was heated at 100 °C for 20 hrs. The reaction mixture was cooled to room temperature and extracted with CH_2Cl_2. The organic layer was washed with water and brine. The organic layer was dried over Na_2SO_4. After filtration, the solvent was evaporated *in vacuo*. The crude product was purified with column chromatography (silica gel / CH_2Cl_2) and recycling preparative HPLC (eluent: $CHCl_3$) to obtain as white solid. Yield: 90%. ^{1}H NMR (400.13 MHz, $CDCl_3$) δ (ppm): 7.64-7.74 (3H, m, Ar*H*), 7.39 (1H, t, J = 8.4 Hz, Ar*H*), 6.68 (2H, d, J = 8.4 Hz, Ar*H*), 3.77 (6H, s, -OCH_3). ^{13}C NMR (100.16MHz, $CDCl_3$) δ (ppm): 157.3, 141.0, 136.7, 131.6, 117.8, 116.1, 115.3, 113.8, 104.2, 55.9. HR-APCI-TOF-Mass (positive) found *m/z*: 265.0976, calcd. for $C_{16}H_{12}N_2O_2$ *m/z*: 265.0972 $[M+H]^+$. FT-IR (ATR) v/ cm^{-1}: 2233 (-CN).

3-(2',6'-di(hexyloxy)phenyl)phthalonitrile: This compound was synthesized from 3-iodophthalonitrile and 2,6-dihexyloxyphenyl boronic acid[1] according to the same procedure of **3**. Yield: 75%. ^{1}H NMR (400.13 MHz, $CDCl_3$) δ (ppm): 7.63-7.75 (3H, m, Ar*H*), 7.39 (1H, t, J = 8.4 Hz, Ar*H*), 6.68 (2H, d, J = 8.4Hz, Ar*H*), 3.75-3.95 (4H, m, -OCH_2-). HR-APCI-TOF-Mass (positive) found *m/z*: 405.2474, calcd. for $C_{26}H_{32}N_2O_2$ *m/z*: 405.2464 $[M+H]^+$. FT-IR (ATR) v/ cm^{-1}: 2234 (-CN).

3,6-bis(2',6'-dimethoxyphenyl)phthalonitrile **4**: A flask containing 3,6-di(triflate)phthalonitrile (50 mg, 0.12 mmol), 2,6-dimethoxybenzeneboronic acid (54 mg, 0.30 mmol), K_3PO_4 (82 mg, 0.48 mmol), $Pd(OAc)_2$ (1.3 mg, 5.9 μmol) and SPhos (3.6 mg, 8.9 μmol) was filled with Ar. 1.2 mL of dry THF was added to the mixture with syringe through septum. The mixture was heated at 70 °C for one night. The reaction mixture was cooled to room temperature and extracted with CH_2Cl_2. The organic layer was washed with water and brine. The organic layer was dried over Na_2SO_4. After filtration, the solvent was evaporated *in vacuo*. The crude product was purified with silica gel column chromatography (eluent: CH_2Cl_2) and recrystallization from CH_2Cl_2/n-hexane to obtain as white solid. Yield: 40%. ^{1}H NMR (400.13 MHz, $CDCl_3$) δ (ppm): 7.62 (2H, s, Ar*H*), 7.39 (2H, t, J = 9.6 Hz, Ar*H*), 6.68 (4H, d, J = 8.4Hz, Ar*H*), 3.81 (6H, s, -OCH_3). ^{13}C NMR (100.16MHz, $CDCl_3$) δ (ppm): 157.5, 138.6, 135.5, 131.1, 117.9, 116.1, 114.2, 104.2, 55.9. HR-APCI-TOF-Mass (positive) found *m/z*: 401.1541, calcd. for $C_{24}H_{20}N_2O_2$ *m/z*: 401.1496 $[M+H]^+$. FT-IR (ATR) v/ cm^{-1}: 2233 (-CN)

1,8,15,22-tetra(2',6'-dimethoxyphenyl)phthalocyanine **5**: Li metal (16 mg, 2.3 mmol) was dissolved in dry 1-hexanol (2.5 mL) at 70 °C under Ar. After Li was perfectly dissolved, the mixture was cooled down to room temperature. **3** (110 mg, 3.8 mmol) was added to this mixture. The reaction mixture was heated at 157 °C for 5 hrs under Ar. After cooling to r.t, few drops of acetic acid was added to the reaction mixture and was diluted with methanol. The precipitate was collected with filtration and residue was washed with methanol for several times. The crude product was purified with column chromatography (Al_2O_3 / CH_2Cl_2). Yield: 48% MALDI-TOF-Ms (dithranol) found *m/z*: 1058.6, calcd. for $C_{64}H_{50}N_8O_8$ *m/z*: 1058.4 [M^+].

1,4,15,18-tetra(2',6'-dimethoxyphenyl)phthalocyanine **6**: Li metal (2.8 mg, 0.40 mmol) was dissolved in dry 1-hexanol (0.35 mL) at 70 °C under Ar. After Li was perfectly dissolved, the mixture was cooled down to room temperature. Phthalonitrile (32 mg, 0.25 mmol) and **4** (50 mg, 0.125 mmol) were added to this mixture. The reaction mixture was heated at 160 °C for 4 hrs under Ar. After cooling to r.t, few drops of acetic acid was added to the reaction mixture and was diluted with methanol. The precipitate was collected with filtration and residue was washed with methanol for several times. The crude product was purified with column chromatography (Al_2O_3, eluent: CH_2Cl_2/n-hexane = 5:1) and recycling preparative HPLC (eluent: $CHCl_3$). Yield: 3% ^{1}H NMR (400.13 MHz, $CDCl_3$) δ (ppm): 8.54 (4H, s, Ar*H*), 7.96 (4H, s, Ar*H*), 7.82 (4H, t, J = 8.4 Hz, Ar*H*), 7.08 (8H, d, J = 8.4 Hz, Ar*H*), 3.60 (24H, s, -OC*H*$_3$), -0.52 (2H, s, -N*H*). HR-APCI-TOF-Mass (positive) found *m/z*: 1059.3996, calcd. for $C_{64}H_{50}N_8O_8$ *m/z*: 1059.3824 $[M+H]^+$.

1,8,15,22-tetra(2',6'-hydroxyphenyl)phthalocyanine **7**: **6** (47 mg, 44.2 μmol) was dissolved in CH_2Cl_2 (1.2 ml) at 0 °C. 1 mol/L CH_2Cl_2 solution of BBr_3 (0.53 ml, 0.53 mmol) was added to this solution and stirred at room temperature for 8 hrs. After the addition of few drops of water, the resulting precipitate was filtered and dried in *vacuo*. Yield: 62%. MALDI-TOF-Ms (dithranol) found *m/z*: 948.2, calcd. for $C_{56}H_{34}N_8O_8$ *m/z*: 948.2 [M^+]. FT-IR (ATR) *ν*/ cm^{-1}: 3300 (-OH).

1,4,15,18-tetra(2',6'-hydroxyphenyl)phthalocyanine **8**: **8** was synthesized by the same procedure of **7**. Yield 75%. MALDI-TOF-Ms (dithranol) found *m/z*: 948.6, calcd. for $C_{56}H_{34}N_8O_8$ *m/z*: 948.2 [M^+]. FT-IR (ATR) *ν*/ cm^{-1}: 3300 (-OH).

1,8,15,22-tetra(2',6'-hexyloxyphenyl)phthalocyanine **1**: **6** (8.0 mg, 8.4 μmol) was dissolved in dry DMF (38 ml). 1-Bromohexane (45 mg, 0.27 mmol) and dry K_2CO_3 (37

mg, 0.27 mmol) were added to this solution and heated at 60 °C for 3 days. The reaction mixture was cooled to room temperature and extracted with CH_2Cl_2. The organic layer was washed with water and brine. The organic layer was dried over Na_2SO_4. After filtration, the solvent was evaporated *in vacuo*. The crude product was purified with silica gel column chromatography (Al_2O_3 / CH_2Cl_2) and recycling preparative HPLC (eluent: $CHCl_3$). Yield: 66%. ^{1}H NMR (400.13 MHz, $CDCl_3$) δ (ppm): 8.53 (4H, d, J = 8.8Hz, Ar*H*), 7.93-7.98 (8H, m, Ar*H*), 7.70 (4H, t, J = 8.4 Hz, Ar*H*), 7.00 (8H, d, J = 8.4 Hz, Ar*H*), 3.93-3.97 (8H, m, -OCH_2-), 3.74-3.80 (8H, m, -OCH_2-), 0.99-1.03 (16H, m, -CH_2-), 0.21-0.51 (48H, m, -CH_2-), 0.06-0.10 (24H, m, -CH_3), -0.56 (2H, s, -N*H*). ^{13}C NMR($CDCl_3$, 100.62MHz): δ (ppm) = 158.9, 133.7, 129.3, 129.01, 122.5, 119.9, 106.32, 69.0, 31.1, 29.1, 25.4, 22.2, 13.7; HR-APCI-TOF MS *m/z* 1620.0050 [M^-], Calcd for $C_{104}H_{130}N_8O_8$: *m/z* 1620.0039; MALDI-TOF-MS *m/z* 1621.6 $[M+H]^+$.

1,4,15,18-tetra(2’,6’-hexyloxyphenyl)phthalocyanine **2**: **2** was synthesized by the same procedure of **1**. Yield: 33%. ^{1}H NMR (400.13 MHz, $CDCl_3$) δ (ppm): 8.44 (4H, d, J = 5.6Hz, Ar*H*), 8.14 (4H, s, Ar*H*), 7.89 (4H, t, J = 5.6 Hz, Ar*H*), 7.05 (8H, d, J = 8.4 Hz, Ar*H*), 3.90-3.95 (8H, m, -OCH_2-), 3.71-3.76 (8H, m, -OCH_2-), 0.99-1.03 (16H, m, -CH_2-), 0.21-0.51 (48H, m, -CH_2-), 0.06-0.10 (24H, m, -CH_3), -0.51 (2H, s, -N*H*). ^{13}C NMR($CDCl_3$, 100.62MHz): δ (ppm) = 157.48, 138.56, 135.54, 131.10, 117.89, 116.07, 114.21, 104.18, 55.86. HR-APCI-TOF MS *m/z* 1620.0080 [M^-], Calcd for $C_{104}H_{130}N_8O_8$: *m/z* 1620.0039; MALDI-TOF-MS *m/z* 1621.1 $[M+H]^+$.

Reference

1. I. Sircar, K. S. Gudmundsson, R. Martin, J. Liang, S. Nomura, H. Jayakumar, B. R. Teegarden, D. M. Nowlin, P. M. Cardarelli, J. R. Mah, S. Connell, R, C. Griffith, E. Lazarides, *Bioorg. Med. Chem.*, **2002**, *10*, 2051-2066.
2.

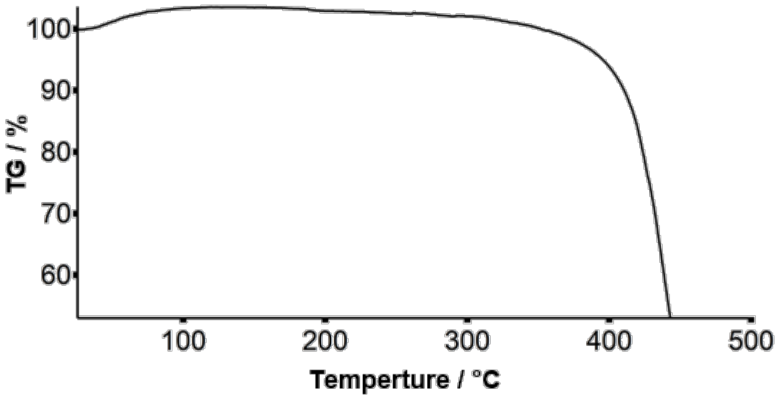

Fig. S1 TGA profile of **1** under N_2 (scan rate 10 °C/min).

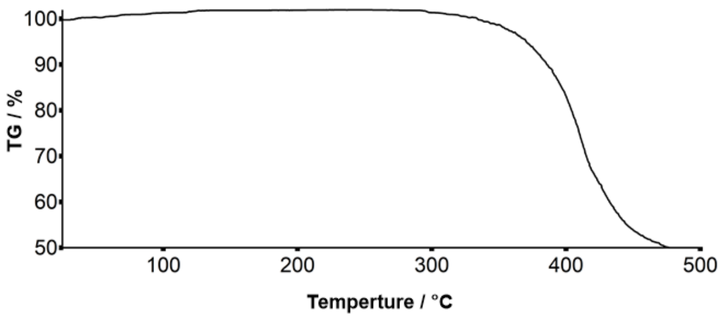

Fig. S2 TGA profile of **2** under N_2 (scan rate 10 °C/min).

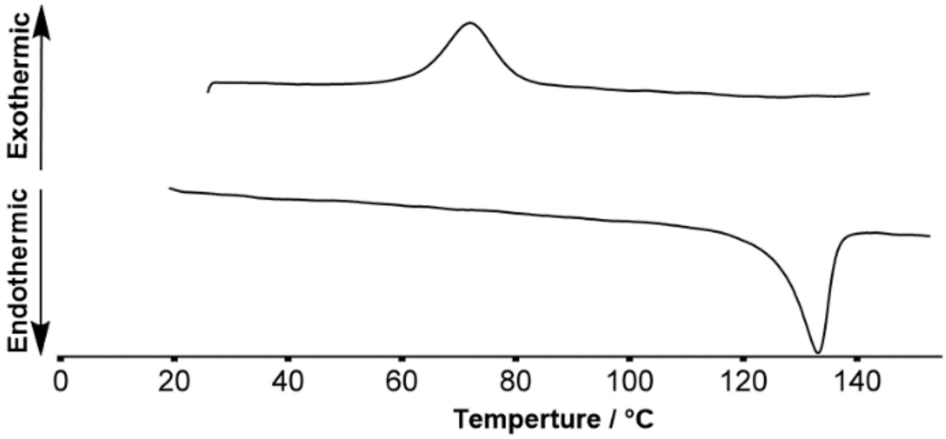

Fig, S3 DSC thermogram of **1** during the first cooling (top line) and the second heating scans (bottom line) at 10 10 °C/min.
